# Adult Rat Motor Neurons Do Not Re-Establish Electrical Coupling during Axonal Regeneration and Muscle Reinnervation

**DOI:** 10.1371/journal.pone.0123576

**Published:** 2015-04-13

**Authors:** Morgana Favero, Alberto Cangiano, Giuseppe Busetto

**Affiliations:** 1 Department of Neurological and Movement Sciences, Section of Physiology and Psychology, University of Verona, 37134, Verona, Italy; 2 National Institute of Neuroscience, 37134, Verona, Italy; Instituto Murciano de Investigación Biosanitaria-Virgen de la Arrixaca, SPAIN

## Abstract

Gap junctions (GJs) between neurons are present in both the newborn and the adult nervous system, and although important roles have been suggested or demonstrated in a number of instances, in many other cases a full understanding of their physiological role is still missing. GJs are expressed in the rodent lumbar cord at birth and mediate both dye and electrical coupling between motor neurons. This expression has been proposed to mediate: (i) fast synchronization of motoneuronal spike activity, in turn linked to the process of refinement of neuromuscular connections, and (ii) slow synchronization of locomotor-like oscillatory activity. Soon after birth this coupling disappears. Since in the adult rat regeneration of motor fibers after peripheral nerve injury leads to a recapitulation of synaptic refinement at the target muscles, we tested whether GJs between motor neurons are transiently re-expressed. We found that in conditions of maximal responsiveness of lumbar motor neurons (such as no depression by anesthetics, decerebrate release of activity of subsets of motor neurons, use of temporal and spatial summation by antidromic and orthodromic stimulations, testing of large ensembles of motor neurons) no firing is observed in ventral root axons in response to antidromic spike invasion of nearby counterparts. We conclude that junctional coupling between motor neurons is not required for the refinement of neuromuscular innervation in the adult.

## Introduction

Gap junctions (GJs) establish communication between proximate cells through channels called connexons, in turn resulting from the assembly of 12 subunits called connexins, that let the passage of electrical currents, small molecules and dyes (for a recent review see [1]). In most regions of the CNS, coupling is transiently expressed during a particular period of development and declines sharply during maturation [2]. An extreme variety of sites in excitable tissues (nervous and muscle systems) and of roles have been described or proposed for GJs. They encompass functions after injury, during development, communications involving glial cells and mechanisms of electrical coupling taking part in the normal firing properties of neurons and of muscle fibers (for example mediating the normal spread of excitation in cardiac myocytes).

Here we concentrate on the role of GJs in the development of the neuromuscular system. GJs have been shown to be expressed in motor neurons of newborn mammals, transiently mediating both dye and electrical coupling [3–5]. Some gap junctional coupling has also been shown to be re-expressed among axotomized adult motor neurons [6]. These events are of considerable interest for the development of muscle innervation perinatally and of reinnervation in the adult, specifically for the process of synapse competition and elimination, the link being the timing of action potential firing in motor neurons and in their motor endings on muscle fibers [7,8]. We therefore undertook an investigation of the possible electrical coupling between motor neurons after axotomy in adult rats, in non-anesthetized decerebrate preparations with large motoneuronal populations examined, and special attention to the firing activity of these neurons in response to electrical coupling.

## Materials and Methods

### Animals and surgery

Procedures for animal experiments were authorized by the Istituto Superiore di Sanita’ and the Ministry of Health of Italy. Adult male Wistar rats, 200–350 g (Harlan), were used for two groups of experiments: one was designed to trace the time course of leg muscle reinnervation after nerve damage, while the other group was used to test whether lumbar motor neurons become electrically coupled during the reinnervation process. In both groups the left sciatic nerve was crushed with fine forceps at its exit from the iliac bone, under Equithesin anesthesia (9.7 mg/mL sodium penthobarbital, 42.5 mg/mL chloral hydrate, administered i.p. at 0.2–0.4 mL 100 g^-1^ body weight). The rats were returned to their cages and recovered from the anesthesia showing complete paralysis of the left leg. After surgery, rats were carefully monitored every day and did not show obvious signs of distress: body weight was comparable to that of rats of same age and strain, they kept their fur clean, had no eye, nose and ear secretions, their breath rate and depth were normal and they normally interacted with the littermates and explored the environment. The surgical scar in no case developed signs of infection and rats showed minimal pain-related reaction by touching it during the first days after surgery. For these reasons we did not treat them with antibiotics or painkillers. At different days after the crush (14 through 32), those of the first group (10 rats) were anesthetized again with Equithesin to record muscle contractions following indirect and direct electrical stimulation, i.e. of nerve and muscle respectively. The left leg was introduced into a chamber filled with modified Ringer solution [9] equilibrated with 95% O_2_ and 5% CO_2_, and the temperature kept at 36°C with a local heater. Distal tendons of soleus, extensor digitorum longus (EDL) and tibialis anterior (TA) muscles were sequentially connected to a strain gauge (model FT03E, Grass Instruments, U.S.A.) for isometric tension recording. Supramaximal rectangular voltage pulses (50 μs, stimulator: Master-8, A.M.P.I., Israel and custom-made S.I.U) were delivered through suction electrodes to the distal stump of common peroneal nerve (for EDL and TA) and posterior tibial (for soleus). Direct stimuli (600 μs) were applied through two chlorided silver plates closely flanking the muscle, from a constant current generator (Basile, Italy) supplying up to 300 mA, while D-tubocurarine (Wellcome) was present in the bathing solution (20 mg l^-1^) to evoke only direct, maximal responses. Twitch and tetanic contractions were recorded at optimal length for twitch (amplifier: CyberAmp 320 and TL-1 A/D Interface; acquisition: Axotape software; all from Axon Instruments, U.S.A.). Trains of stimuli eliciting tetanic contractions were 1 s in duration and maximal tension was measured at the best frequency for the muscle.

**Fig 1:**
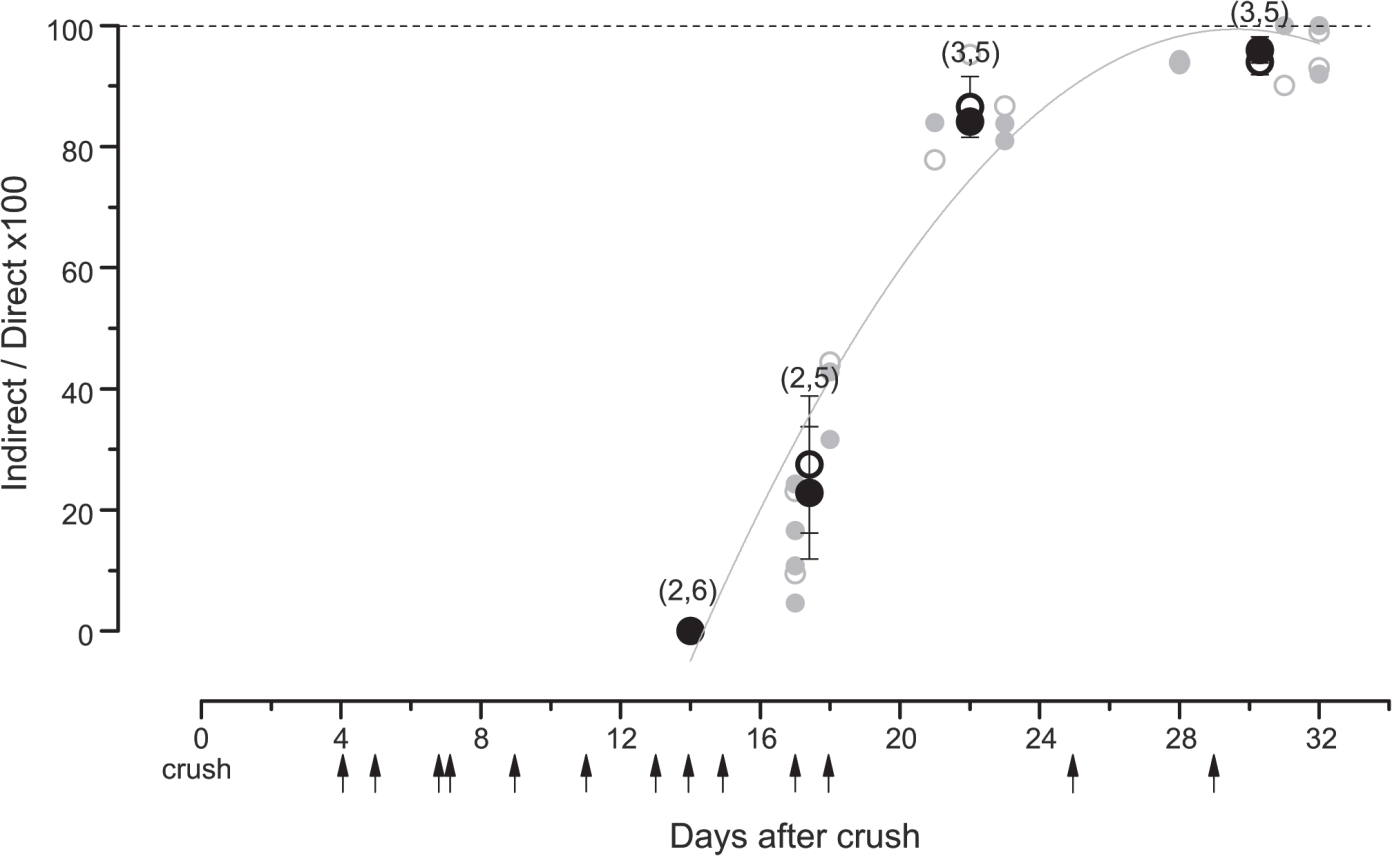
Time course of rat hindlimb muscle reinnervation after crush of the sciatic nerve. The percent reinnervation is obtained by comparing maximal twitch and tetanic isometric contractions elicited by nerve (indirect) electrical stimulation with those obtained, under curare, by muscle (direct) stimulation. The muscles investigated are soleus, extensor digitorum longus (EDL) and tibialis anterior (TA), from a series of 10 rats, utilized only for determining the time course of reinnervation. Filled and open gray dots (twitch and tetanus, respectively) are the individual data from all muscle types. Filled and open black dots (twitch and tetanus, respectively) represent the average (± SE) at different time points of reinnervation after crush (in brackets: number of rats, number of muscles). The gray line is the polynomial fitting curve of individual twitch data (r = 0.97; that for tetanus data, also with r = 0.97, not shown).The arrows under the abscissa indicate the times after crush when we investigated, in another series of animals, the possible development of electrical coupling between motor neurons during regeneration of their axons.

As regards the second group of rats (n = 13), at different days after the crush (5 through 29, as indicated by the arrows under the abscissa in Fig 1) under deep anesthesia through spontaneous breathing of air with ether, we inserted a tracheal cannula, ligated the internal carotid arteries and introduced a polyethylene cannula in the femoral vein. We then made a laminectomy to uncover the lumbar spinal cord with its dorsal and ventral roots; finally we opened the skull and transected the midbrain at the intercollicular level; in 2 rats we also ablated the anterior lobe of the cerebellum. After quick hemostasis of the bleeding at the base of the skull, we closed the skin wound over the head and discontinued the ether anesthesia. The animal was then kept warm at 36–37°C with a custom-made electrical blanket with feedback control from a rectal thermometer. The animal spontaneous breathing remained completely normal and, after rapid recovery from anesthesia, developed a pronounced rigidity of the 4 legs, as expected, so that they could stand upright if properly supported to maintain balance [10,11]. After not less than 1 hour from interruption of the ether anesthesia, we mounted the rat in a spinal frame (Narishige, Japan), raised the skin flaps over the lumbar spinal cord to form a pool that was filled with paraffin oil.

At this stage we connected a respiratory pump (Harvard Apparatus, U.S.A.) to the tracheal cannula, injected i.v. curare (pancuronium bromide, 30 μg 100 g^-1^, Sigma) to block movements, due to the high reactivity of the un-anesthetized decerebrate state of our preparations. We also monitored the EKG activity through a custom-made amplifier. We then cut the dorsal roots (DRs) L3 through S2 near their dorsal root ganglia (DRG) on the left side (that of the sciatic nerve crush), isolated the cut DRs L4 and L5, as well as the left ventral roots (VRs) L4 and L5 albeit leaving them initially intact. Finally we dissected free the previously crushed sciatic nerve at mid thigh level. These procedures were also done in 3 rats whose sciatic nerve had not been crushed, serving for control purposes.

### Electrophysiological experiments

**Fig 2:**
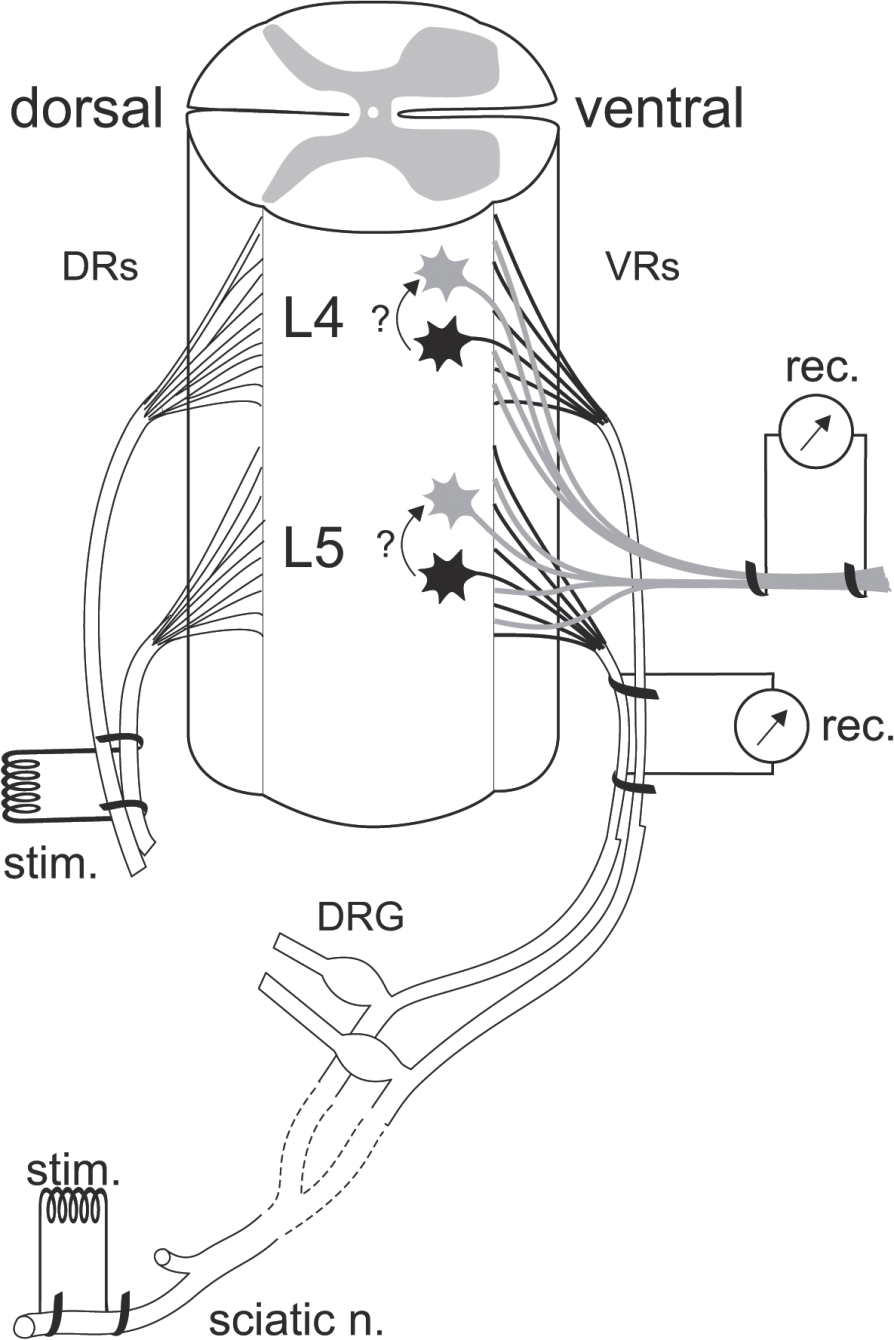
Schematic diagram illustrating the preparation used for the acute electrophysiological experiment. Dorsal roots (DRs), ventral roots (VRs), dorsal root ganglia (DRG) and the lumbar spinal cord are outlined. After intercollicular decerebration, the ether anesthesia is discontinued and a laminectomy exposes the lumbar cord, DRs L3 through S2 are cut, DRs L4 and L5 are mounted on stimulating electrodes, and finally VRs L4 and L5 are isolated and mounted on diphasic recording electrodes. A tiny fascicle of VR axons is initially cut and diverted on monophasic recording electrodes. After a series of antidromic (sciatic nerve) and orthodromic (DRs) stimulations are performed, while recording from VR axons both diphasically and monophasically, a further tiny bundle of axons is shifted to the monophasic recording electrodes and the stimulation series repeated. This procedure is reproduced about 10 times until most, although not all, VR axons are diverted to the monophasic recording electrodes. The cell bodies in the ventral horn and the axons of the motor neurons invaded antidromically (from spikes elicited in the sciatic nerve) are represented in black, while those *possibly* receiving an electrically-transmitted depolarization and their axons are represented in gray. Arrows and question marks depict the possible electrical coupling of motor neurons.

Refer to Fig 2 for the following description. First, the left sciatic nerve was inserted in a silicone cuff containing 2 stimulating stainless steel wires. On the left side, the DRs L4 and L5 were mounted on stimulating electrodes. The corresponding VRs were mounted on recording electrodes: from the outset, however, a very small bundle of axons was cut away and diverted on another set of recording electrodes (called ventral rootlet), with the distal electrode being placed near the cut+crushed end. The proximal electrode rested on the rootlet at about 1 cm of distance from the distal one. This way, we obtained a diphasic record from the undivided portion of VRs L4 an L5, both of the orthodromic response to DRs L4-L5 stimulation and of the antidromic response to sciatic nerve stimulation. But we also obtained clear-cut monophasic responses to DRs L4-L5 stimulation (mono and polysynaptic reflexes) and a *possible* orthodromic response of motor neurons evoked by the antidromic volley through electrical coupling (amplifier: CyberAmp 320, Axon Instruments, U.S.A.; acquisition: SPIKE 2 software, C.E.D., U.K.). After a complete series of antidromic and orthodromic stimulations, we repeated several times the transfer of a small bundle of motor axons, trying to obtain an optimum input/output ratio between the antidromically invaded motor neurons and the possibly electrically coupled ones. For antidromic and orthodromic stimulations we used both single shocks and trains of up to 5 shocks, alone and combined, as explained in detail in the Results. Single shocks were rectangular pulses of 50 μs in duration.

Trying to modulate gap junctional conductance, we also explored changing pH and temperature. For the first we used respiratory alkalosis and acidosis varying the ventilation level of the respiratory pump (as GJ channels can be modulated by pH: see [12,13]) while for the second we changed the temperature of the oil pool, as detailed in the Results.

## Results

### Time course of leg muscle reinnervation after sciatic nerve crush

The aim of the present work was to determine whether electrical coupling between rat motor neurons, transiently present during the first few weeks of postnatal life, is re-expressed in the adult life when their axons regenerate, following damage, and re-establish their peripheral connections. As this possible event could again occur transiently, we had to know at which delay times after axonal crush (performed at the sciatic nerve exit from the iliac bone), we should test the presence or absence of motoneuronal electrical coupling in acute electrophysiological experiments. Our guide was the time course of reinnervation of various hindlimb muscles by branches of the sciatic nerve. We conducted this preliminary investigation in a devoted group of animals and at appropriate days after nerve crush, in order to determine the start- and the end-time point of reinnervation. All animals were sacrificed after having collected the recordings of muscle contraction evoked by nerve and muscle stimulations (see Materials and Methods for details). Fig 1 shows the data relative to soleus, EDL and TA muscles of the reinnervated side, and indicates that they are all reinnervated essentially at the same time, from start to completion, given a similar distance from the site of the crush. This distance is of the order of several centimeters, thus imposing quite a few days for the regenerating axons to reach their muscular targets. After a delay of ~14 days, reinnervation begins and is essentially complete in a further ~14 days, as also shown in detail previously [14,15]. Fig 1 also shows averages obtained by pooling together the measurements from different muscles and different animals recorded at the beginning or at the end of the process. The arrows under the abscissa indicate the time when we performed the acute electrophysiological experiments to test electrical coupling, in a different series of animals, also after sciatic nerve crush, each animal being again sacrificed at the end of the acute experiment. Thus we performed this testing at different levels of progression of axonal regeneration, from early times when the targets have not yet been reached, to completion of target reinnervation.

### Search for electrical coupling between lumbar motor neurons, during adult regeneration of their axon

We planned the acute electrophysiological experiment in order to increase our chances of detecting electrical coupling between motor neurons, should it be re-expressed in the adult condition during regeneration following damage of their axons. To determine whether motor neurons fired action potentials in response to antidromic spike invasion and consequent depolarization via GJs, we selected conditions that could increase their excitability. The first choice was to perform stimulation and recording series without anesthesia, which was made possible by decerebration. This procedure also induces rigidity [10,11], that is depolarization especially of extensor α motor neurons via activation of γ motor neurons and increase in muscle spindle discharge (γ rigidity [12,16]). Potentially, this also increased motor neuron excitability, but we could not fully exploit it because of the partial curarization necessary to prevent movement artifacts during recording, with possible blockade also of γ endplates. However in two rats we also ablated the anterior lobe of the cerebellum, the day of the acute experiment, a procedure that induces a marked direct facilitation of motor neurons (α rigidity [10,12,16]) that would not be affected by the curare blockade.

**Fig 3:**
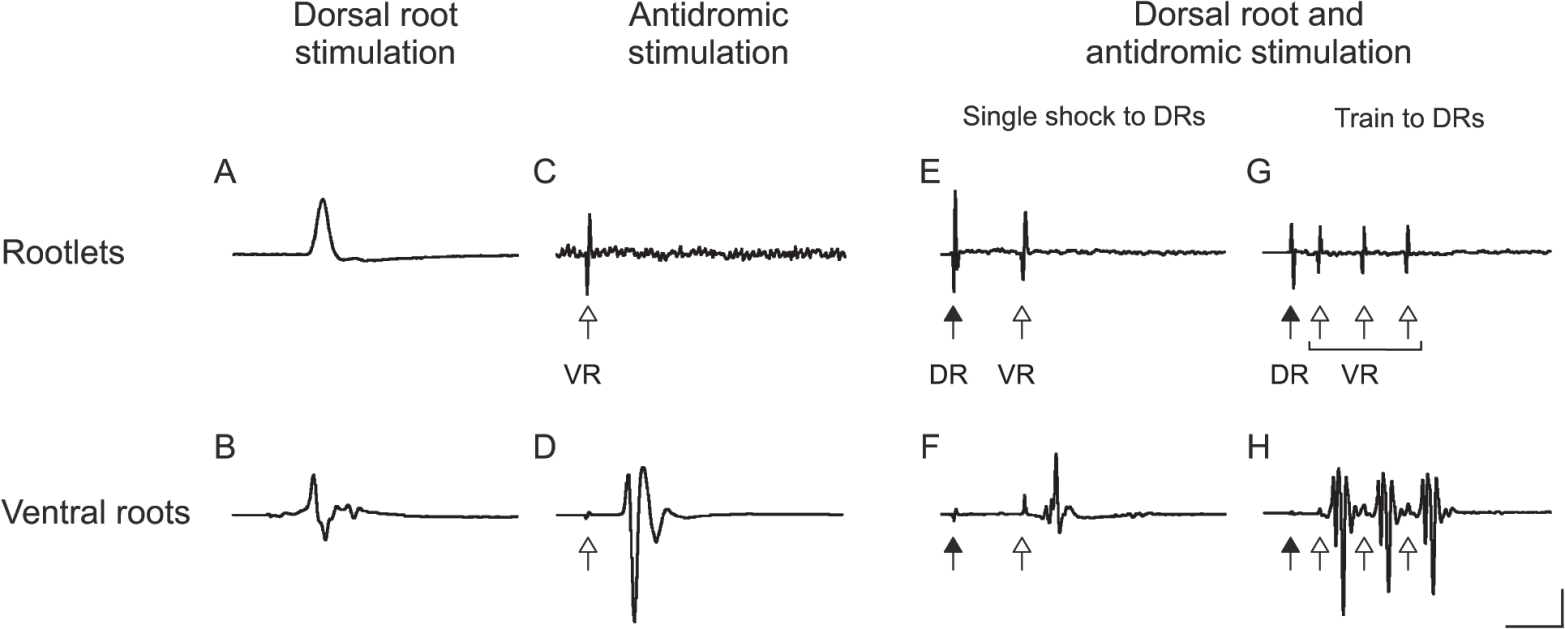
Antidromic action potential invasion of L4 and L5 motor neurons, evoked by stimulation in the sciatic nerve of their regenerating axons, does not lead to firing of nearby motor neurons. This is true either when the antidromic stimulation is acting alone (C,D; VR = ventral root) or when it is combined with conditioning facilitation evoked by stimulation of the dorsal roots (E,F,G,H; DR = dorsal root). For positions of stimulating and recording electrodes refer to Fig 2 and its legend. All traces are averages of 5–10 responses; stimulus artifacts to the dorsal roots are marked by filled head arrows, while those to the sciatic nerve by open head arrows. (A and B) Mono- and polysynaptic compound action potentials of lumbar motor neurons, recorded from cut ventral rootlets (A, monophasic record), or from the remaining intact ventral root axons (B, diphasic record); in both records the stimulus (single shock to the dorsal roots) occurs slightly before the sweep onset (~0.5 ms). (C and D) Although the action potentials evoked by single shocks to the sciatic nerve are, as expected, conducted antidromically to the spinal cord (record of the retrograde incoming volley in D), they fail to excite L4 and L5 motor neurons whose axons are not stimulated and instead recorded monophasically from diverted ventral rootlets as shown in C. Records A,B,C,D are from the same rat 5 days after sciatic nerve crush. (E,F,G,H) The antidromic (test) supramaximal stimulation of the sciatic nerve is combined with conditioning stimulation of the dorsal roots (at strength immediately below threshold for the appearance of reflex VR responses), single or repetitive and at variable intervals as specified in Results. (E,F) A single test shock to the sciatic nerve, following after 5 ms a conditioning single shock to the DRs, evokes an antidromic incoming volley in F, but no visible response after the test stimulus artifact in the rootlets record in E; records E,F are from a rat 17 days after nerve crush. (G,H) A train of 5 conditioning stimuli at 100 Hz to the DRs, precedes by 2 ms a train of three test stimuli at 300 Hz to the sciatic nerve, eliciting three incoming volleys in the ventral roots as seen in H, but no response in the rootlets monophasic record of G (the first stimulus artifact visible in the sweeps G and H, is the last of the five conditioning single shocks to the DRs); records G,H are from a rat 18 days after nerve crush. Voltage calibrations: 750 μV (A,B,D); 50 μV (C,E,G); 150 μV (F,H). Time calibrations: 2 ms (A,B,C,D); 4 ms (E,F,G,H).

A further way to facilitate motoneuronal firing in response to antidromic spike invasion of nearby motor neurons, was temporal and spatial summation (for positions of stimulating and recording electrodes refer to Fig 2 and its legend). For temporal facilitation of coupling potentials we used repetitive retrograde stimulation supramaximal for VR axons using trains of up to 5 stimuli at frequencies of 100, 200 and 300 Hz (electrically transmitted potentials successfully follow high frequency activation [17]), since reports of depolarizing coupling potentials in newborn rats indicate a time course of ~20 ms [3–5]. Moreover, another obvious way we used to induce a background excitation of motor neurons, were monosynaptic reflex excitatory responses, elicited by stimulation of the central stump of transected DRs of the same L4 and L5 spinal segments whose motor neurons were tested by antidromic spike invasion. Single or repetitive stimuli were used for this conditioning orthodromic facilitation. For repetitive stimulation we used again trains of up to 5 stimuli at frequencies of 50 and 100 Hz. The net effect of stimulating myelinated axons in the DRs, was to elicit mono- and polysynaptic responses in the VR axons of both the monophasic and the diphasic recordings (Fig 3A and 3B respectively), the latency of the peak monosynaptic component with respect to a single shock to the DRs averaging in several rats ~3.0 ms (n = 7). In Fig 3B the sharp first peak of the diphasic monosynaptic response is preceded by several wavelets, likely representing the transmission of the field potential of incoming volleys along primary afferents and of synaptic potentials in motor neurons and interneurons. We elicited these reflex mono- and polysynaptic responses in all rats (13 experimental and 3 controls), and their amplitudes were comparable to those shown in Fig 3A and 3B, except in one experimental animal that gave much smaller responses, due to partial damage of the spinal cord during laminectomy, and was discarded. Unlike retrograde stimulation, the strength used to elicit this conditioning orthodromic facilitation was kept immediately below that sufficient for the appearance of the smallest monosynaptic response in the VRs: this was necessary to detect the possible response to test antidromic stimulation without contamination from orthodromically evoked potentials. Summing up, conditioning DR and test VR stimulations were based on combinations of single shocks and/or trains of up to 5 shocks, the maximum number of stimuli applied in a given run being 5 in a conditioning train and 5 in a test train. Based on records of reflex responses and antidromic volleys, we estimated that the invasion of the motor neuron cell bodies by orthodromically and antidromically evoked depolarization occurs after comparable delays (~2.5 ms). Accordingly, we varied the time interval between conditioning and test single shock stimuli in a range most frequently comprised in a few ms in both directions, seldom reaching a maximum of ±20 ms. When trains were used, this interval was that between the last stimulus of a conditioning train (or a single conditioning shock) and the first stimulus of a test train (or a single test shock).

A final way we systematically used to find an optimal in/out ratio for the electrical coupling between antidromically invaded and nearby motor neurons, was to progressively divert small groups of axons from the intact VRs to monophasic recording electrodes (as shown in Fig 2), and each time repeat a full series of antidromic and orthodromic stimulations.

The complex series of stimulations thus performed in 12 experimental rats at different times over the entire time course of regeneration of sciatic nerve axons up to full muscle reinnervation and in 3 control rats, amounted to over one thousand trials. The results are however simple to describe because in no instance did an antidromic volley elicit a response in the monophasically recorded rootlets, even at high amplification (examples in Fig 3C, 3E and 3G). Yet there was no doubt about a normal functioning of the spinal cord circuitry, as assured by the always present mono- and polysynaptic responses of motor neurons (Fig 3A and 3B) as well as the full sized antidromic volleys (Fig 3D, 3F and 3H). In particular, the maximal amplitude of the monosynaptic response measured when all axons had been diverted from the initially intact L4 or L5 VRs to the monophasic recording electrodes (Fig 2) was always large (1.6 ± 0.29 mV on average, n = 7 roots from 6 animals) and in accordance with the amplitude previously described for similarly non anesthetized rodent preparations [18–21].

In the records of the intact ventral root axons we often observed, quite some time after the incoming retrograde α motor volleys, small wavelets whose shortest latency was 6.0 ms (average of 5 rats; examples in Fig 3F and 3H): they could be either antidromic spikes conducted at lower velocity than those of α motor fibers, that is γ fiber spikes, or orthodromic spikes backfiring from some of the antidromically invaded α motor neurons (similar to the F wave of the H reflex: see [12,22]). They could certainly not be potentials due to electrical coupling, for the following reasons: 1) they would be expected to occur at a much earlier time (estimated latency, after the stimulus artifact to the sciatic nerve, 3–3.5 ms), 2) they are not observed in the monophasic record of the rootlets in 3 of 5 cases in which the late wavelets were recorded in the intact ventral root axons; in the 2 positive cases, the late wavelets disappeared from the rootlet record after cutting dorsal root S1 (which had escaped section in the initial preparation for the acute experiment) thus showing their nature of late polysynaptic reflexes.

In a small number of rats we also attempted to modulate GJ conductance by changing pH (see [12,13]) through alkalosis by hyperventilation of up to 5 min in 5 rats or acidosis by hypoventilation of up to 2 min in 1 rat. We also tried to increase the responsiveness of motor neurons by changing the temperature of the oil pool over the spinal cord (4 rats, range 18–40°C), as lowering the temperature is known to increase the excitability of neurons including motor neurons [23]. Also with these procedures we did not obtain indications of electrical coupling following antidromic spike invasion of L4 and L5 motor neurons.

## Discussion

The main result of this study is that no overt signs of electrical coupling, namely firing of lumbar motor neurons in response to antidromic spike invasion of adjacent motor neurons, are detected during adult axonal regeneration. In fact, following the demonstration of the transient expression of GJs and electrical coupling in newborn rodents [3–5], we asked whether this is recapitulated in adults after nerve injury and repair, as do many other developmental features of the neuromuscular system. It is true that a similar attempt made in adult cats only detected the re-expression of dye coupling but not of electrical coupling after axotomy [6]. However, here we examined this question in conditions of maximal responsiveness of the spinal circuitry involved. Moreover, as a measure of functionally relevant levels of re-coupling we explored if it could be strong enough to drive motor neurons to fire, because this would have direct implications for the synchronizing effects that are discussed in detail later.

The use of decerebrated non-anesthetized preparations greatly increased the level of spontaneous excitation of motor neurons: all animals displayed clinical signs of rigidity [10,11] and the central nervous system, including the spinal cord circuitry, was not depressed by anesthetics [24]. In addition, as explained in the Results, we performed extracellular recordings from ventral roots so that we could obtain information from a large number of motor neurons. Moreover, we took advantage of temporal and spatial summation to enhance the likelihood that even a weak coupling had a measurable effect, especially in experiments causing the interaction between orthodromic and antidromic depolarization of motor neurons. The importance of testing the influence of orthodromic activation, becomes particularly clear by recalling that action potentials with a dendritic origin, absent in normal motor neurons, develop after axotomy in adult cat motor neurons thus markedly enhancing synaptic transmission [25]; also relevant in this connection is that gap junctional coupling would occur, particularly in adults, at dendritic locations [26], and thus produce, if alone (i.e. not integrated with synaptic potentials), little or even no effect at the soma. The progressive shift of bundles of motor axons from the antidromically stimulated to the orthodromically recorded VRs, during the acute experiment, tested a broad range of input/output relationships within a potentially coupled motor neuron population. As a further proof of maximal responsiveness of the spinal circuit, the monosynaptic reflex response recorded monophasically was always large and comparable to that described by others in non-anesthetized preparations [18–21]. Finally, we also attempted to uncover the possible presence of silent (i.e. non conducting) gap junctions by changing pH, by hyper- or hypoventilation, as pH is known to be a modulatory factor [12,13].

In spite of all these efforts the results were consistently negative, prompting considerations relevant for the physiological role of GJs in several ways.

First, synchronization of neuronal firing is one of the physiological consequences of electrical coupling. In the case of motor neurons, a transient synchronization has been observed during the developmental refinement of their connections on muscle fibers: a tight correlation of action potential firing between spontaneously active motor neurons has in fact been recorded in vivo in behaving rats during the first days of postnatal life [27] and in the in vitro mouse spinal cord at P0-P2 [28]. One is not dealing with a simple correlation of firing of the *discharges* of different motor neurons (see [29], reporting a broad central peak of cross-correlation of 1–3 sec width), which is a natural consequence of the fact that neurons of the same motor pool fire in concert at all ages, in smaller or greater numbers, to evoke contraction of the target muscle. Instead, the time correlation detected is between *individual spikes* of different motor neurons, the duration of the peak of cross-correlation being of the order of a few tens of ms (5–25 ms in [27]; 30–100 ms in [28]). During the first week of postnatal life, firing of motor neurons of the same pool undergoes rapid de-synchronization in vivo [27], which is the stable, prevalent, adult condition [16,30]. Data for the contribution of GJs between motor neurons to their correlated firing in newborn rodents has been reported [29,31,32], although the type of correlation examined in these studies is slow in character, as pointed out above. On the other hand, another study reported that the GJ blocker carbenoxolone does not significantly affect the tight, fast correlation of firing observed at P0-P2, in doses effective in antagonizing the electrically mediated short-latency coupling potential evoked by antidromic VR stimulation [28]. The latter study strongly suggests the existence during development of a “common drive” impinging on motor neurons, whereby synchronicity is already organized at the pre-motor neuron level without a determinant contribution of GJs (see also [33–37]).

There are some well established facts about the physiological role of the sequence synchronization-desynchronization described in the previous paragraph: 1) the transition to desynchronization appropriately precedes in time [27] the process of synapse elimination that occurs in muscle fibers perinatally. In fact, starting from the innervation by several motor neurons of each muscle fiber that characterizes the embryonic innervation (polyneuronal innervation), a rapid elimination occurs in early postnatal life leading to the innervation by a single collateral of a motor neuron [7,8,38,39], the permanent adult condition; 2) the time course of the fast correlation of firing is linked to the process of polyneuronal innervation/synapse elimination, as shown by the following findings: (a) synchronous activation of inputs, induced in vivo in adult rats by electrical stimulation during synapse elimination, exerts a clearcut inhibitory action on the elimination process [40,41]; (b) an equal amount of imposed activity, but with an asynchronous paradigm, instead strongly promotes elimination [42]. The entire phenomenon can be understood as an activity-dependent process of hebbian competition between multiple inputs on every target myofiber, each trying to remain the exclusive input of that fiber ([8] for a recent review).

As far as the adult model of polyneuronal innervation/synapse elimination is concerned, which is obtained by a peripheral nerve injury [41, 43–44], we are not aware that any study has documented that the axotomized motor neurons transiently resume the high correlation of firing which is the hallmark of newborn animals. Should synchronization occur, the present study would indicate that “common drive” may be responsible, rather than re-expression of GJs or positive modulation of their conductance. However, should synchronization not occur, the adult polyneuronal innervation would still be understood as a process distinctive of the formation of new synaptic contacts on muscle fibers, precisely as in the embryo; and the subsequent synapse elimination would be explained as activated by the asynchronous firing of adult motor neurons, again like it has been shown to occur in newborn animals [27]. Actually, lack of an initial *synchronous* firing would be consistent with the much lower amount of polyneuronal innervation seen in adult with respect to newborn animals [41].

Why, then, are dye and electrical coupling transiently expressed in newborn animals? It is possible that this is one instance of the redundant mechanisms often seen in development, contributing, together with common drive, to the synchronization of slow locomotor oscillations. Or, alternatively, it may serve developmental functions based on the exchange of chemical signals, instead of electrical currents: namely the proposed role of gap junction communication in the developmental specification of neuronal identity and circuit formation [2].

Another relevant issue to be dealt with here is the possible participation of GJs to the production of motor behavior in adult mammals [28,45]. Synchronous DC membrane potential oscillations of many motor neurons recorded simultaneously, after block of chemical transmission by removing extracellular Ca^2+^ or block of action potentials with tetrodotoxin, have been observed in the isolated spinal cord of newborn mice at P0-P2, during the NMDA-induced “fictive locomotion” [28,45] (see also [46]). GJs mediate this slow synchronization, because it is prevented by the GJ blocker carbenoxolone. The possibility that such a participation of GJs to the generation of motor output in the immature mammalian spinal cord may extend to the adult animal has been raised [47], even though GJ de-coupling has been repeatedly reported to occur following the first few days after birth and to persist in the adult life [3–5] (with the known exception of a motor pool related to sexual function [48]). The attractiveness of this proposal was compounded by: 1) the widespread expression of connexins and junctional plaques in the adult mammalian spinal cord, albeit they are more prevalent in early development, and 2) the fact that gap junction communication can be modulated through various mechanisms [49]. Certainly, the fact that a strong stimulus such as axotomy does not induce VR firing in response to antidromic invasion, as shown here in a context of marked motoneuronal facilitation, does not make very likely the occurrence of a re-coupling of adult motor neurons, even via a modulatory mechanism. But it is still possible that in specific contexts, like the production of motor behavior, a dynamic recruitment of adult GJs may occur [47]. Finally, it is appropriate to recall here what we mentioned earlier, i.e. that in mice at P0-P2 there is also a much faster synchronization of motor neuron spikes [27, 28], which is not blocked by carbenoxolone, and is certainly in good part mediated by common inputs from interneurons [28]. It may appear intriguing that although GJs are present and functional in the newborn, since they mediate the slow locomotor-like synchronization, they do not appear essential for the fast spike synchronization. However, there are two likely explanations for this: 1) the slow oscillations are recorded during block of chemical transmission and of spike activity, so that the GJ coupling remains the only driver of the synchronization between motor neurons, and 2) the long buildup of activity during the expression of these oscillations allows sufficient time for a progressive locking in phase of the motor neuron population, even if their mutual strength of coupling is relatively weak.
